# Supplementary material for: Assessing Knowledge, Attitudes and Behaviours toward Salt and Sugar Consumption in the Central Division of Fiji
Source: Nutrients. 2024 Sep 28;16(19):3288. doi: 10.3390/nu16193288 (PMC11478911; doi:10.3390/nu16193288)
Supplement: Supplementary file 1 [file nutrients-16-03288-s001.zip › nutrients-3174988-supplementary.pdf]

Knowledge, Attitudes and Behaviours Questionnaire
Fiji Household Survey

INTRODUCTION

The next questions ask about your knowledge, attitudes and behaviour towards dietary salt and sugar. Dietary salt includes ordinary table salt, unrefined salt such as sea salt, iodized salt and salty sauces such as soya sauce or ketchup (see showcard). Dietary sugar includes raw and white sugar (including sugar lumps), brown sugar, cane sugar, caster and icing sugar. The following questions are on adding salt and sugar to the food right before you eat it, on how food is prepared in your home, on eating processed foods that are high in salt such as *breads, instant noodles, tinned and processed meats or sauces*, and sugar such as *cakes, biscuits and fizzy drinks* and questions on controlling your salt and sugar intake. Please answer the questions even if you consider yourself to eat a diet low in salt and sugar.

DIETARY SALT QUESTIONS

Behaviours

1. In your household, do you normally do the cooking?

☐ Yes
☐ No

- If "no" go to question 2b.

2a. When you are cooking, how much salt or salty sauces such as soy sauce do you normally add? (USE SHOWCARD)

☐ More than a teaspoon of salt/ more than a tablespoon of salty sauces
☐ A teaspoon of salt/ a tablespoon of salty sauces
☐ Less than a teaspoon of salt/ less than a tablespoon of salty sauces
☐ I don't add salt or salty sauces when cooking
☐ Don't know

2b. How much salt or salty sauces such as soy sauce do you add to your meal before eating? (USE SHOWCARD)

☐ More than a teaspoon of salt/ more than a tablespoon of salty sauces
☐ A teaspoon of salt/ a tablespoon of salty sauces
☐ Less than a teaspoon of salt/ less than a tablespoon of salty sauces
☐ I don't add salt or salty sauces when cooking
☐ Don't know

3. How often do you add salt or salty sauce such as soy sauce to your food right before you eat it or as you are eating it?

☐ Always (approximately 100% of the time)
☐ Often (≥50% of the time)

☐ Sometimes (<50% of the time)
☐ Rarely (≤20% of the time)
☐ Never (0% of the time)
☐ Don't know

4. How often do you eat processed food high in salt? (By processed food high in salt, I mean foods that have been altered from their natural state, such as packaged salty snacks, canned salty food including pickles and preserves, salty food prepared at a fast food restaurant, cheese, processed meat)

☐ Always (every day in the week)
☐ Often (4-5 days in the week)
☐ Sometimes (3-4 days in the week)
☐ Rarely (2-3 days in the week)
☐ Never (none of the days in the week)
☐ Don't know

5a. Do you try to reduce your salt intake?
☐ Yes
☐ No
☐ Don't know

- If "no" go to question 6.

5b. If you answered "Yes" to question 5, can you indicate how you try to reduce your salt intake? (select as many as relevant)

- Limit consumption of packaged processed foods
- Limit consumption of take-aways/fast food
- Look at the salt/sodium content on food labels
- Buy low salt alternatives
- Use spices other than salt when cooking
- Other
    - If other please describe:

Attitudes
6. How important to you is lowering the salt in your diet?
☐ Very important
☐ Somewhat important
☐ Neutral (don't have an opinion on it being important or not important)
☐ Somewhat unimportant
☐ Not at all important
☐ Don't know

Knowledge

7a. What do you think is the recommended amount of salt that you should eat each day? (USE SHOWCARD)

☐ Less than 10g
☐ Less than 5g
☐ Less than 2g
☐ Don't know

7b. Based on your answer to question 7a. what does this look like? (interviewer to show display card, depicting teaspoon amounts of salt)

☐ Two teaspoons of salt
☐ One teaspoon of salt
☐ Half a teaspoon of salt
☐ Don't know

8. Do you think that consuming too much salt or salty sauce can lead to any of the following health conditions? (tick all that apply)

- Hypertension
- Stroke
- Osteoporosis
- Diabetes
- None of the above
- Don't know

DIETARY SUGAR QUESTIONS

Behaviours

1a. How often do you drink drinks that you add sugar to (hot or cold e.g. coffee, tea, milo/hot chocolate, water, juice)?

☐ Daily
☐ 5-6 days per week
☐ 3-4 days per week
☐ 1-2 days per week
☐ 1-3 days per month
☐ Less than once a month
☐ Never (0 days a week)

- If you answered "never" go to question 2

1b. How much sugar do you add to your drinks (hot or cold e.g. coffee, tea, milo/hot chocolate, water, juice)?

☐ A tablespoon of sugar
☐ 3 teaspoons of sugar

☐ 2 teaspoons of sugar
☐ 1 teaspoon of sugar
☐ Less than a teaspoon of sugar
☐ Don't know

2a. How often do you drink sugar sweetened beverages (e.g. fizzy drinks, sodas, juice, raro/concentrate)?
☐ Daily
☐ 5-6 days per week
☐ 3-4 days per week
☐ 1-2 days per week
☐ 1-3 days per month
☐ Less than once a month
☐ Never (0 days a week)

- If you answered "never" go to question 3

2b. If you drink sugar sweetened beverages, how much would you normally drink in total over a day?
☐ Equivalent of a 1 litre bottle or more
☐ A 500mL bottle
☐ A can (330mL)
☐ A cup (250 mL)
☐ Less than a cup
☐ Don't know

3. Do you try to reduce your sugar intake?
☐ Yes
☐ No
☐ Don't know

- If you answered "no" go to question 5

4. If you answered "Yes" to question 4, can you please indicate how you try to reduce your sugar intake?

- Limit consumption of packaged processed foods
- Limit consumption of sugar sweetened beverages
- Limit the addition of sugar to hot or cold drinks
- Limit use of instant drink mixes (e.g. coffee mixers)
- Limit consumption of confectionary
- Limit consumption of baked goods, like cakes and sweet biscuits, sweet pastries, and ice cream
- Buy low sugar alternatives
- Other
    - If other, please describe:

Attitudes

5. How important to you is lowering the sugar in your diet?
⊡ Very important
⊡ Somewhat important
⊡ Neutral (don't have an opinion on it being important or not important)
⊡ Somewhat unimportant
⊡ Not at all important
⊡ Don't know

Knowledge
6a. What do you think is the recommended amount of sugar that you should eat each day?
⊡ Less than 20% of total energy intake
⊡ Less than 10% of total energy intake
⊡ Less than 5% of total energy intake
⊡ Don't know

6b. Based on your answer to question 6. what does this look like? (interviewer to show display card, depicting teaspoon amounts of sugar)

7. Do you think that consuming too much sugar or sugar sweetened beverage can lead to any of the following health conditions? (tick all that apply)
- Diabetes
- Obesity
- Heart disease
- High cholesterol
- Poor dental health
- None of the above
- Don't know
